# Author Correction: Pattern invariance for reaction-diffusion systems on complex networks

**DOI:** 10.1038/s41598-020-60111-5

**Published:** 2020-02-18

**Authors:** Giulia Cencetti, Pau Clusella, Duccio Fanelli

**Affiliations:** 1 Università degli Studi di Firenze, Dipartimento di Ingegneria dell’Informazione, Florence, Italy; 2 Università degli Studi di Firenze, Dipartimento di Fisica e Astronomia and CSDC, Florence, Italy; 3 INFN Sezione di Firenze, Florence, Italy; 4 Institute for Complex Systems and Mathematical Biology, SUPA, University of Aberdeen, Aberdeen, UK

Correction to: *Scientific Reports* 10.1038/s41598-018-34372-0, published online 01 November 2018

The Acknowledgements section in this Article was omitted. The Acknowledgements section should read:

“This work has been funded by the EU’s Horizon 2020 research and innovation programme under the Marie Skłodowska-Curie Grant Agreement No. 642563.”

