# Supplementary material for: Pattern invariance for reaction-diffusion systems on complex networks
Source: Sci Rep. 2018 Nov 1;8:16226. doi: 10.1038/s41598-018-34372-0 (PMC6212431; doi:10.1038/s41598-018-34372-0)
Supplement: Supplementary file 1 — Supplementary Information [file 41598_2018_34372_MOESM1_ESM.pdf]

Pattern invariance for reaction-diffusion systems on complex networks

Giulia Cencetti,[1,2,3] Pau Clusella,[4,2] and Duccio Fanelli[2,3]

[1] *Università degli Studi di Firenze, Dipartimento di Ingegneria dell'Informazione, Florence, Italy*
[2] *Università degli Studi di Firenze, Dipartimento di Fisica e Astronomia and CSDC, Florence, Italy*
[3] *INFN Sezione di Firenze, Italia*
[4] *Institute for Complex Systems and Mathematical Biology, SUPA, University of Aberdeen, Aberdeen, UK*

I. DERIVATION OF DISPERSION RELATION

Let us consider a generic system composed of N identical entities linked through a complex network. At time t the activity of node j is described by an m-dimensional variable $\boldsymbol{w}_j(t) \in \mathbb{R}^m$. Starting from a specific initial state $\boldsymbol{w}_j(0)$, the dynamics of $\boldsymbol{w}_j$ evolves according to

$$\dot{\boldsymbol{w}}_j = \boldsymbol{\mathcal{F}}(\boldsymbol{w}_j) + K\sum_{k=1}^{N} A_{jk}\boldsymbol{\mathcal{G}}(\boldsymbol{w}_k - \boldsymbol{w}_j) \qquad j = 1, \ldots, N. \tag{S1}$$

Let us consider the general case where the variable $\boldsymbol{w}_j$ is a m-dimensional vector. By perturbing the homogeneous solution and inserting $\boldsymbol{w}_j = \boldsymbol{w}^* + \boldsymbol{\delta w}_j$ in equation (S1) we obtain

$$\dot{\boldsymbol{\delta w}}_j = \boldsymbol{\partial_w \mathcal{F}}(\boldsymbol{w}^*)\boldsymbol{\delta w}_j + K\boldsymbol{\partial_w \mathcal{G}}(\boldsymbol{0})\sum_{k=1}^{N}\Delta_{jk}\boldsymbol{\delta w}_k = \sum_{k=1}^{N} J_{jk}\boldsymbol{\delta w}_k \tag{S2}$$

where $\boldsymbol{\partial_w \mathcal{F}}(\boldsymbol{w}^*)$ and $\boldsymbol{\partial_w \mathcal{G}}(\boldsymbol{0})$ are m-dimensional matrices of derivatives. The Jacobian matrix $\boldsymbol{J} = (J_{jk})$ has dimension $Nm \times Nm$. It is however possible to convert $\boldsymbol{J}$ in a block-diagonal matrix by changing basis to the one provided by Laplacian eigenvectors. We then diagonalize the Laplacian, $\sum_k \Delta_{jk}\phi_k^{(\alpha)} = \Lambda^{(\alpha)}\phi_j^{(\alpha)}$, and express the perturbation on the new basis:

$$\boldsymbol{\delta w}_j(t) = \sum_{\alpha=1}^{N}\boldsymbol{c}_\alpha(t)\phi_j^{(\alpha)}, \tag{S3}$$

where $\boldsymbol{c}_\alpha \in \mathbb{R}^m$. Consequently, (S2) can be decoupled:

$$\sum_{\alpha=1}^{N}\dot{\boldsymbol{c}_\alpha}(t)\phi_j^{(\alpha)} = \boldsymbol{\partial_w \mathcal{F}}(\boldsymbol{w}^*)\sum_{\alpha=1}^{N}\boldsymbol{c}_\alpha(t)\phi_j^{(\alpha)} + K\boldsymbol{\partial_w \mathcal{G}}(\boldsymbol{0})\sum_{\alpha=1}^{N}\Lambda^{(\alpha)}\boldsymbol{c}_\alpha(t)\phi_j^{(\alpha)} \tag{S4}$$

and using the linear independence of the eigenvectors, it is reduced to a set of N m-dimensional systems indexed by α,

$$\dot{\boldsymbol{c}_\alpha}(t) = \boldsymbol{J}_\alpha \boldsymbol{c}_\alpha(t) \tag{S5}$$

where the $m \times m$ matrix $\boldsymbol{J}_\alpha \equiv \boldsymbol{\partial_w \mathcal{F}}(\boldsymbol{w}^*) + K\boldsymbol{\partial_w \mathcal{G}}(\boldsymbol{0})\Lambda^{(\alpha)}$ is the α-th diagonal block of $\boldsymbol{J}$. At this point a distinction becomes necessary. If the Jacobian is constant in time, a simple solution of (S5) takes the form $\boldsymbol{c}_\alpha(t) = \boldsymbol{c}_\alpha(0)e^{\lambda^{(\alpha)}t}$ and it is straightforward that in order to admit a non-trivial solution of (S5) the determinant of $\boldsymbol{J}_\alpha - \lambda^{(\alpha)}\mathbb{1}_m$ must be zero. From this condition we obtain $\boldsymbol{\lambda}^{(\alpha)} = (\lambda_k^{(\alpha)})$ which configures as the set of eigenvalues of the block $\boldsymbol{J}_\alpha$.
If instead the Jacobian is periodic in time due to the limit cycle solution ($\boldsymbol{J}_\alpha(t+T) = \boldsymbol{J}_\alpha(t)$), the Floquet theory [1–3] comes into play. This latter allows us to express again the temporal dependence of the perturbations $\boldsymbol{\delta w}$ as an exponential function: $e^{\mu_k^{(\alpha)}t}$, where $\mu_k^{(\alpha)}$ are the *Floquet exponents*, defined by $\mu_k^{(\alpha)} = \log(\rho_k^{(\alpha)})/T$ with $\rho_k^{(\alpha)}$ eigenvalues of the constant matrix $\boldsymbol{B}$ such that $\det(\boldsymbol{B}) = \exp\left[\int_0^T tr(\boldsymbol{J}_\alpha(t))dt\right]$.
In both, the static and the periodic case, the stability of the system is assessed by the component of respectively $\boldsymbol{\lambda}^{(\alpha)}$ or $\boldsymbol{\mu}^{(\alpha)}$ with maximum real part and its relation with the Laplacian eigenvalues is called the *dispersion relation.*

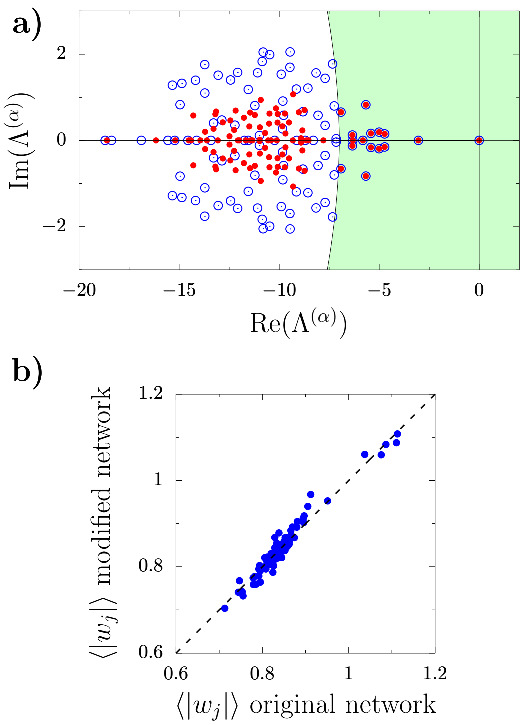

Figure S1: (a): Original (red open circles) and modified (blue closed circles) eigenvalues in the complex plane, the coloured area corresponds to the instability region. (b): patterns correlation, corresponding to $R^2 = 0.95$.

II. EIGENMODE RANDOMIZATION FOR DIRECTED NETWORKS

The procedure outlined in the main text requires a special attention when applied to a system where the spatial support is a directed network. The main difference lies in the asymmetry of the Laplacian matrix, so that the eigenvalues and eigenvectors are, in general, complex. In this case, the unstable eigenmodes can be identified by observing how the eigenvalues are placed in the complex plane. A instability region, corresponding to the positive part of the dispersion relation, can be delineated following the procedure explained in the main text, as it is represented in Fig. S1. The eigenvalues appearing in this region correspond to the eigenmodes that compose the unstable manifold, which should be preserved to obtain similar patterns in a secondly generated network. All the other eigenvalues and eigenvectors can be modified so as to obtain a new Laplacian matrix. Let us recall that the eigenvalues $\Lambda^{(\alpha)}$, due to the fact that the original Laplacian matrix is real, either are real or are complex and come in conjugate pairs. The same happens for the components of the eigenvectors $\boldsymbol{\phi}^{(\alpha)}$. These characteristics have to be preserved during the randomization in order to obtain a meaningful spectrum for the new Laplacian. However, if the eigenvector modification is done by using a rotation matrix, the new eigenvectors $\tilde{\boldsymbol{\phi}}^{\alpha}$ will not, in general, preserve the complex conjugate couples, which happen to be randomly rotated in the $(N-n)$-dimensional space. This ultimately implies that the imaginary parts do not mutually cancel out and that the entries of a Laplacian obtained from

$$\tilde{\boldsymbol{\Delta}} = \tilde{\boldsymbol{\Phi}}\tilde{\boldsymbol{\Lambda}}\tilde{\boldsymbol{\Phi}}^{-1}$$

would be complex. Let us however observe that cutting away the imaginary part of $\tilde{\boldsymbol{\Delta}}$, we obtain a third version of the Laplacian, $\hat{\boldsymbol{\Delta}} = \mathrm{Re}(\tilde{\boldsymbol{\Delta}})$, whose eigenmodes can still serve to our aim. In fact, the new eigenvectors and eigenvalues either are real or complex conjugate and in particular, the n eigenmodes of the original Laplacian to be left invariant are still preserved. This can be proven by observing that the Laplacian $\tilde{\boldsymbol{\Delta}}$, even if it is complex, is built so as to correctly maintain the unstable manifold, and its imaginary part (which is eliminated in defining $\hat{\boldsymbol{\Delta}}$) only involves the stable eigenmodes. Therefore, cutting out the imaginary part of $\hat{\boldsymbol{\Delta}}$ translates into a second randomization of the stable eigenmodes, that does not affect the unstable manifold where the pattern information is eventually stored. In

order to prove this statement we should consider the imaginary part of a generic (i,l) Laplacian entry:

$$(\tilde{\Delta}_{\rm Im})_{il} = \sum_{\alpha=1}^{N} \tilde{\Lambda}^{\alpha}_{\rm Im}[(\tilde{\Phi}_{\rm Re})_{i\alpha}(\tilde{\Phi}^{-1}_{\rm Re})_{\alpha l} - (\tilde{\Phi}_{\rm Im})_{i\alpha}(\tilde{\Phi}^{-1}_{\rm Im})_{\alpha l}] + \tilde{\Lambda}^{\alpha}_{\rm Re}[(\tilde{\Phi}_{\rm Re})_{i\alpha}(\tilde{\Phi}^{-1}_{\rm Im})_{\alpha l} + (\tilde{\Phi}_{\rm Im})_{i\alpha}(\tilde{\Phi}^{-1}_{\rm Re})_{\alpha l}]. \tag{S6}$$

where $\tilde{\mathbf{\Phi}}_{\rm Re}$ and $\tilde{\mathbf{\Phi}}_{\rm Im}$ are the real and the imaginary part of the eigenvectors. The sum over α can be separated into two sums: one over $\alpha = 1, ..., n$, the other over $\alpha' = n+1, ..., N$, the first containing all the unmodified eigenmodes, the second the randomized ones. We shall prove that the first sum is equal to zero. Let us begin by observing that for index α up to n, the eigenvalues $\tilde{\Lambda}$ and eigenvectors $\tilde{\phi}$ appearing in the sum can be replaced by the original Λ and ϕ. Considering a conjugate couple labeled with β and γ, we have $\Lambda^{(\gamma)} = (\Lambda^{(\beta)})^*$, with

$$\begin{aligned}(\phi_{\rm Re})_{i\gamma} &= (\phi_{\rm Re})_{i\beta}\\ (\phi_{\rm Im})_{i\gamma} &= -(\phi_{\rm Im})_{i\beta}\end{aligned} \tag{S7}$$

and

$$\begin{aligned}(\phi^{-1}_{\rm Re})_{\gamma l} &= (\phi^{-1}_{\rm Re})_{\beta l}\\ (\phi^{-1}_{\rm Im})_{\gamma l} &= -(\phi^{-1}_{\rm Im})_{\beta l}.\end{aligned} \tag{S8}$$

This implies the cancellation in pairs of the terms corresponding to complex conjugate couples. For what concerns the remaining terms, corresponding to real eigenvalues and eigenvectors, it is clear that all the terms in (S6) are automatically zero (remember that $\boldsymbol{\phi}^{-1}_{jl} = \boldsymbol{\phi}_{lj}$). This proves the claim.

In Fig. S1(a) the eigenvalues of an original random network with $N = 100$ and $\langle k \rangle = 10.2$ are displayed in the complex plane together with those of the modified spectrum. The instability region has been drawn by again using the CGL equation as self-dynamics. Fig. S1(b) shows the correlation between time-average modulus patterns obtained from the original and the network generated from the Laplacian $\hat{\mathbf{\Delta}}$.

III. LOCAL REWIRING: ACCEPTANCE THRESHOLD

The local rewiring method for isodynamic network generation is based on a iterative scheme. Each move consists in removing or adding a link between two random nodes and it is accepted only if the unstable manifold is preserved in the new network. The degree of similarity between new and old eigenmodes is established by means of the error function:

$$E = NE_l + E_q;$$

where

$$E_l = \frac{1}{n}\sum_{\alpha=1}^{n} |\tilde{\Lambda}^{(\alpha)} - \Lambda^{(\alpha)}|^2$$

and

$$E_q = \frac{1}{n}\sum_{\alpha=1}^{n} |\langle \tilde{\boldsymbol{\phi}}^{(\alpha)}, \boldsymbol{\phi}^{(\alpha)} \rangle - 1|^2.$$

The factor N is used to ensure that both terms in the expression of E are of the same order. The change is accepted only if the error function E is smaller than a chosen threshold parameter τ.

IV. SUPPLEMENTARY INFORMATION ON THE PATTERNS FOR THE GINZBURG-LANDAU EQUATION

The panel of Fig. S2 provides supplementary figures relative to the system where the CGL dynamics with $K = 1$, $c_1 = 1$, $c_2 = -3$ is applied to the ER network with $N = 100$ nodes used in section 3 of the main text. Fig. (a) shows the complete dispersion relation of the original system (partially reported also in Fig. 1(b,e) of the main text). The first (smallest in absolute value) eigenvalues identify the unstable modes of the dynamics. In Fig. S2(b) we report the time-averaged modulus of the coefficients $\boldsymbol{c}^{(\alpha)}$ from equation (S3), *i.e.*. the coefficients of $\boldsymbol{w}_j(t)$ expressed in the basis

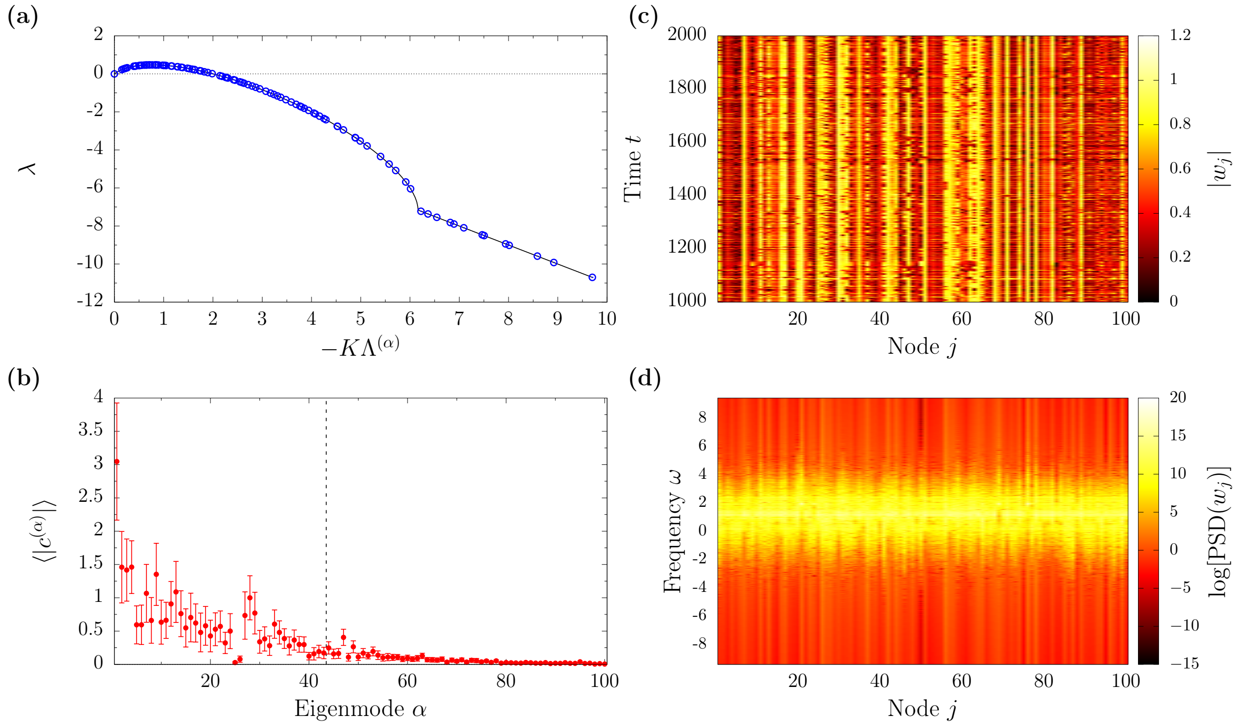

Figure S2: Emergence of spatio-temporal patterns for a Erdös-Rènyi network with $N = 100$ nodes and $\langle k\rangle = 3.5$. **(a)**: Dispersion relation of the network (blue open circles). **(b)**: Time-averaged modulus of the dynamics expressed in the basis of the Laplacian modes. **(c)**: Time evolution of the modulus of each node coded in color. **(d)**: Logarithm of the power spectral density of each node in color code.

of the Laplacian eigenmodes. Thus, it is clear that the modes which are mostly involved into the patterns are those associated to unstable directions. The patterns are shown in Fig. S2(c), obtained as the modulus $|w_j|$ for each node j of the network and for a window of 1000 time steps (same as Fig. 1(g) of the main text). The oscillation frequency (in Fig. S2(d)) of the complex variable w_j does not display relevant information on the network structure, being roughly the same for each node of the network. For this reason we solely refer to the modulus when analyzing the dynamics of the CGL model.

V. ALTERNATIVE SELF-DYNAMICS: BRUSSELATOR MODEL

In order to assess the versatility of the proposed method, we focus on a system which displays a stable homogeneous fixed point, and thus an intrinsically different profile of the associated dispersion relation: the Brusselator model. This system was proposed in 1971 as an example of autocatalytic chemical reaction system [4–6], and is described by the following equations [7]:

$$\begin{cases} \dot{x}_i = a - (b+d)x_i + cx_i^2 y_i + D_x \sum_j \Delta_{ij} x_j \\ \dot{y}_i = bx_i - cx_i^2 y_i + D_y \sum_j \Delta_{ij} y_j \end{cases}$$

with $x_i, y_i \in \mathbf{R}$. This system admits as a solution the homogeneous fixed point $(x_i, y_i) = (\frac{a}{d}, \frac{bd}{ac})\ \forall i$, whose stability depends on the reaction parameters as well as on the underlying network of connections. By using the main text SF reference network and fixing $a = 7$, $b = 10.2$, $c = 1$, $d = 1$, $D_x = 0.1$, and $D_y = 1.4$, the dispersion relation takes the form depicted in Fig. S3(a). The presence of eigenvalues in the positive branch of the curve implies the instability of the uniform fixed point, causing the emergence of irregular patterns.
It is clear that here, differently from the CGL case, the unstable manifold (which contains most of the pattern information) is generated by the modes corresponding to the largest eigenvalues in absolute value. The modes which can be (almost) safely modified in order to generate an isodynamic network consequently correspond to the eigenvalues closest to zero. This amounts to act on the less connected nodes of the network, (see eigenvectors localization in Fig. 3(f) in the main text). The network originated from this procedure will then preserve the tail

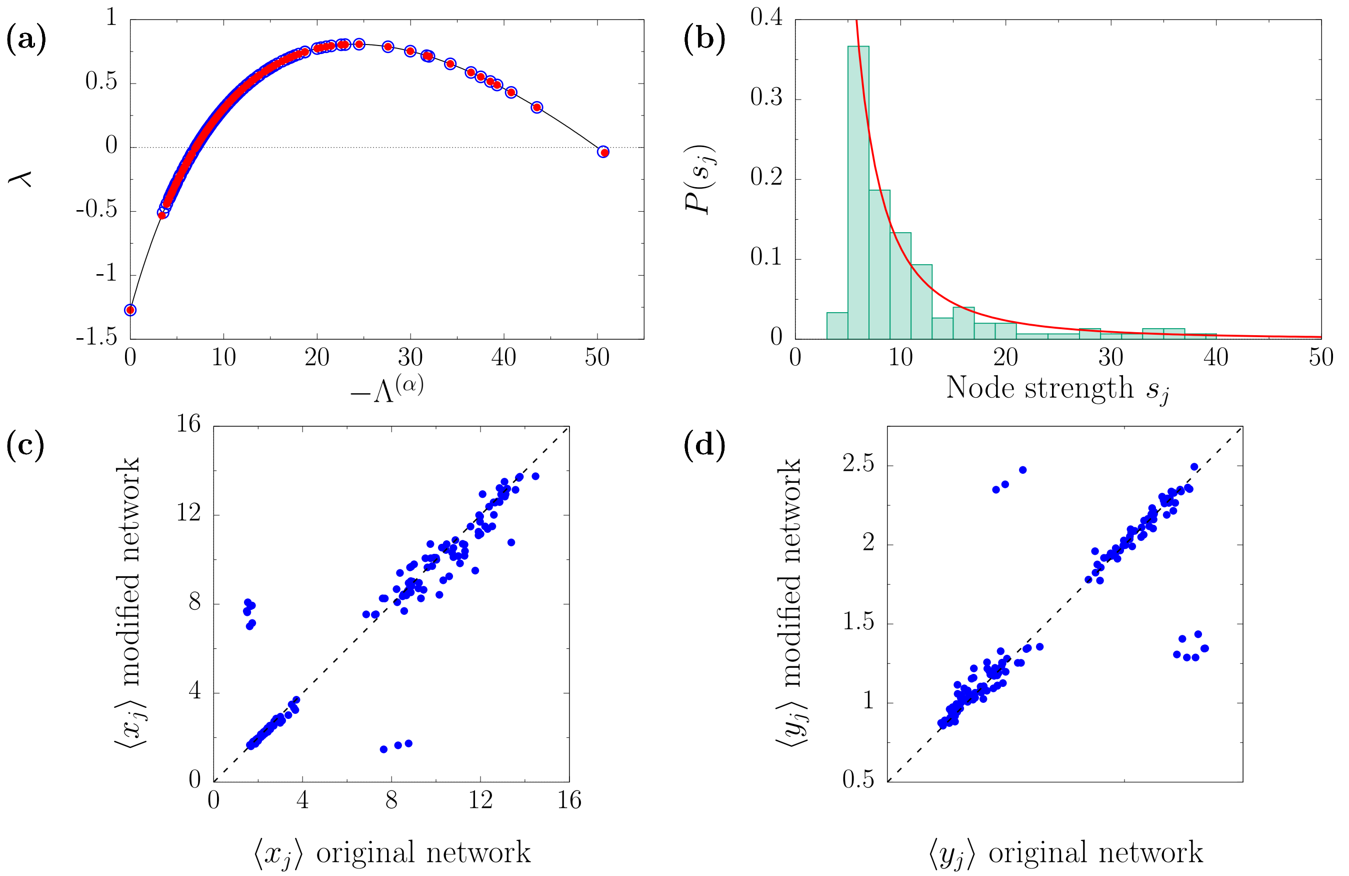

Figure S3: Results obtained from the Brusselator model. (a) Dispersion relation of the original network (blue open circles), and that of the modified network (red small circles). (b) Node strength distribution of the modified network (green boxes) and the corresponding fit to a power law $P(k) = ck^{-\beta}$, with $c = 22.3$ and $\beta = -2.29$ (red curve). (c) Time average activity of x_j for the modified and original networks, the corresponding squared coefficient correlation is $R^2 = 0.85$ (d) Time average activity of y_j for the modified and original networks, with $R^2 = 0.74$. The few clearly out-diagonal points correspond to low connected nodes.

of the characteristic power law shape in the degree distribution, see Fig. 3(c) of the main text and Fig. S3(b) for a comparison between the original $P(k)$ and one realization of the modified version of the network. Figs. S3(c-d) prove the similarity between patterns, as obtained by letting the Brusselator model evolve on the original and on the modified version of the SF network, starting from the same initial conditions.

VI. SUPPLEMENTARY RESULTS ON THE LOCAL REWIRING TECHNIQUE

Figs. S4 and S5 complement Fig. 4 of the main text by adding results corresponding to other realizations of the local rewiring algorithm. The networks obtained using the algorithm do not always provide a good agreement with the patterns obtained from the original network. In fact, depending on the initial perturbation, the degree of correspondence between pattern varies (compare, for instance, Figs. S4(i),(j), and (k)). Nevertheless, it is, in any case, possible to obtain networks that perform largely better than the random rewiring.

On the side of the topology changes, Figs. S4(d),(h),(l) and S5(d),(h),(l) show that most of the modifications involve nodes with a large degree, *i.e.*, those that contribute less to the unstable directions, reflecting the localization properties of the Laplacian eigenvectors (see Fig. 3(d) of the main text).

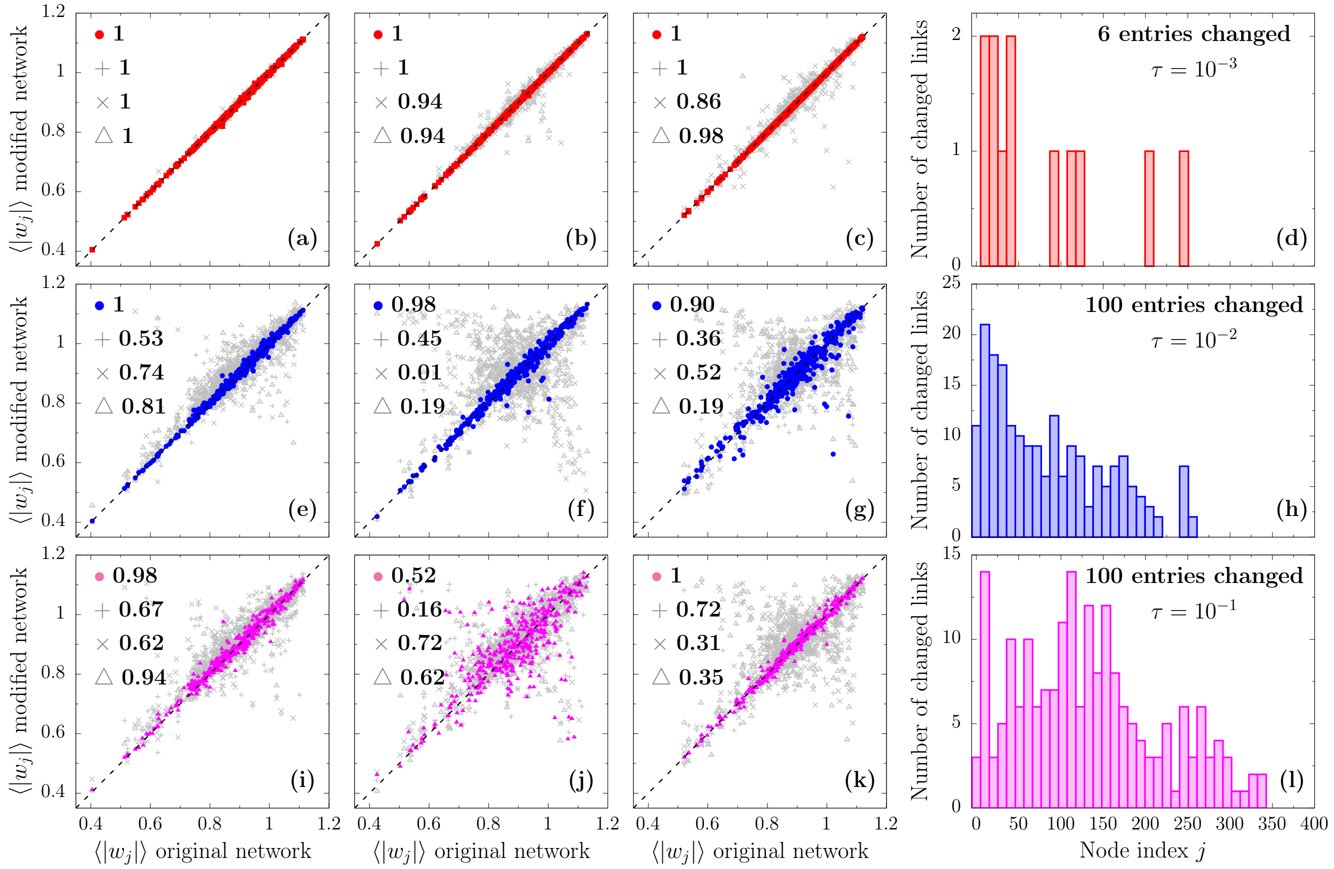

Figure S4: Outcome of the rewiring algorithm for a ER network with 400 nodes and average degree $< k >= 20$. System parameters are $K = 0.15$, $c_1 = 1$, and $c_2 = -3$, for which the system displays 64 unstable modes. (a-c,e-g,i-k) Comparison between the time average modulus of each node in the modified network versus that of the original topology. Each row corresponds to a different modified version of the network: (a-c) consists of a network obtained with $\tau = 10^{-3}$ where 6 links have been changed in the adjacency matrix. (e-g) corresponds to a network obtained with $\tau = 10^{-2}$ where 100 links have been changed. (i-k) corresponds to a network obtained with $\tau = 10^{-3}$ where 100 links have been changed. Each column corresponds to a realization using a different initial condition. Colored circles indicate the results obtained using the local rewiring algorithm, whereas gray plusses, crosses and triangles correspond to a network obtained by changing at random the same number of links. Numbers in the legend indicate the squared correlation coefficient R^2 obtained in each case. (d,h,l) Indicate the number of links changed in for each node of the network, where nodes have been sorted in a descending order according to their degree. Figs. (i) and (l) correspond to Figs. 4(a) and (c) in the main text.

[1] R. Grimshaw, *Nonlinear ordinary differential equations*. Routledge, 2017.
[2] J. D. Challenger, R. Burioni, and D. Fanelli, "Turing-like instabilities from a limit cycle," *Physical Review E*, vol. 92, no. 2, p. 022818, 2015.
[3] M. Lucas, D. Fanelli, T. Carletti, and J. Petit, "Desynchronization induced by time-varying network," *arXiv preprint arXiv:1802.06580*, 2018.
[4] P. Glansdorff and I. Prigogine, "Thermodynamic theory of structure, stability, and fluctuations. 1971."
[5] I. Prigogine and R. Lefever, "Symmetry breaking instabilities in dissipative systems. ii," *The Journal of Chemical Physics*, vol. 48, no. 4, pp. 1695–1700, 1968.
[6] H. Nakao and A. S. Mikhailov, "Turing patterns in network-organized activator-inhibitor systems," *Nature Physics*, vol. 6, no. 7, pp. 544–550, 2010.
[7] T. Biancalani, D. Fanelli, and F. Di Patti, "Stochastic turing patterns in the brusselator model," *Physical Review E*, vol. 81, no. 4, p. 046215, 2010.

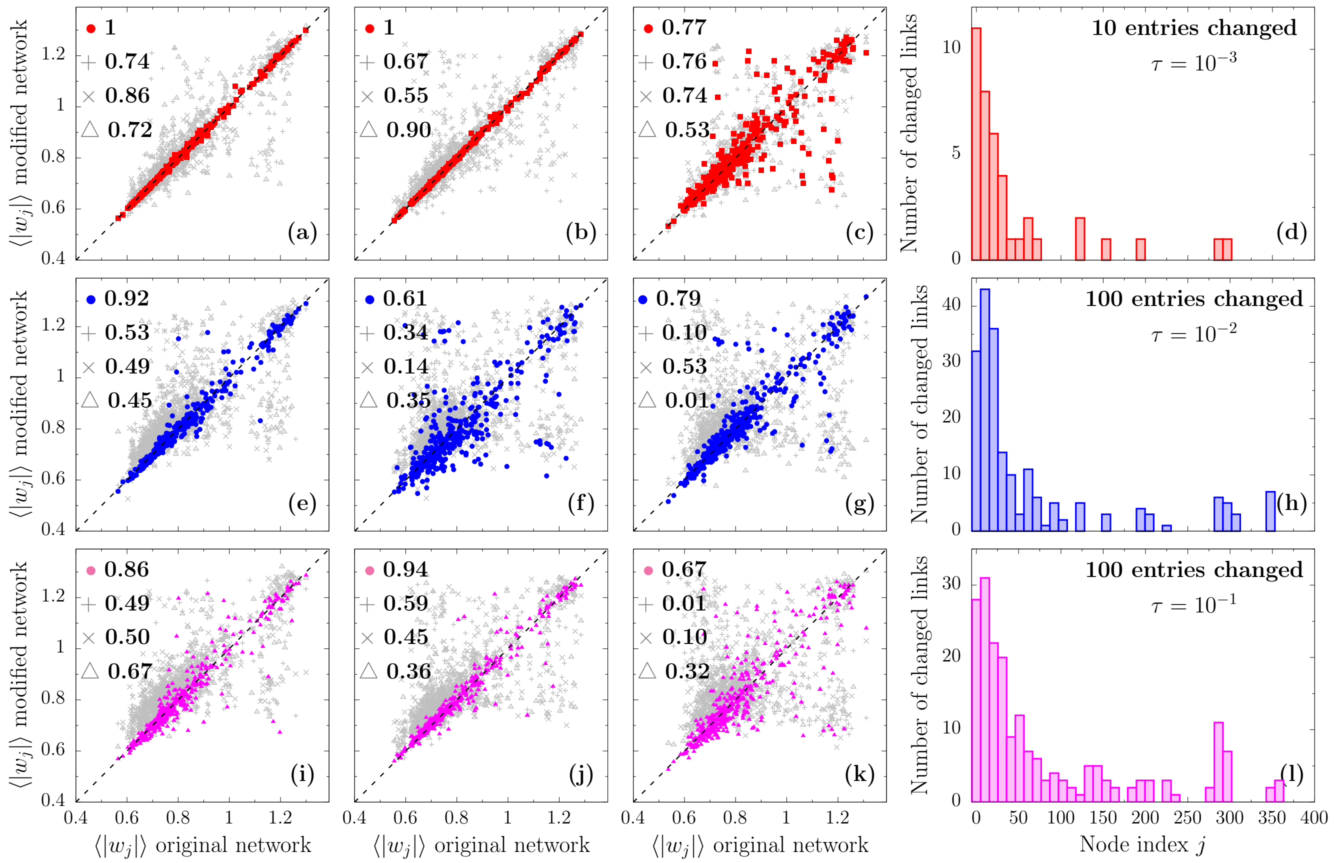

Figure S5: Outcome of the rewiring algorithm for a SF network with 400 nodes. System parameters are $K = 0.376$, $c_1 = 2$, and $c_2 = -2$, which correspond to 63 unstable directions. (a-c,e-g,i-k) Comparison between the time average modulus of each node in the modified network versus that of the original topology. Each row corresponds to a different modified version of the network: (a-c) consists of a network obtained with $\tau = 10^{-3}$ where 10 links have been changed in the adjacency matrix. (e-g) corresponds to a network obtained with $\tau = 10^{-2}$ where 100 links have been changed. (i-k) corresponds to a network obtained with $\tau = 10^{-3}$ where 100 links have been changed. Each column corresponds to a realization using a different initial condition. See Fig. S4 for the legend. (d,h,l) Indicate the number of links changed in for each node of the network where nodes have been sorted in a descending order according to their degree. Figs. (i) and (l) correspond to Figs. 4(b) and (d) in the main text.
